# Supplementary material for: Hydrophobic recognition allows the glycosyltransferase UGT76G1 to catalyze its substrate in two orientations
Source: Nat Commun. 2019 Jul 19;10:3214. doi: 10.1038/s41467-019-11154-4 (PMC6642264; doi:10.1038/s41467-019-11154-4)
Supplement: Supplementary file 1 — Supplementary Information [file 41467_2019_11154_MOESM1_ESM.pdf]

Supplementary Information

Hydrophobic recognition allows the glycosyltransferase UGT76G1 to catalyze its substrate in two orientations

Ting Yang[1]†, Jinzhu Zhang[1]†, Dan Ke[1]†, Wenxian Yang[1]†, Minghai Tang[2], Jian Jiang[1], Guo Cheng[3], Jianshu Li[4], Wei Cheng[2], Yuquan Wei[2], Qintong Li[5]*, James H. Naismith[2,6,7]*, Xiaofeng Zhu[1]*

*** Correspondence and requests for materials should be addressed to X.Z. (email: zhuxiaofeng@scu.edu.cn), J.H.N. (email: naismith@strubi.ox.ac.uk) or Q.L. (email: liqintong@scu.edu.cn)**

† These authors contributed equally: Ting Yang, Jinzhu Zhang, Dan Ke, Wenxian Yang

This file includes

Supplementary Table 1 The synthetic encoding sequence of UGT76G1 and the primers used in this study

Supplementary Tables 2 Data collection and refinement statistics of X-ray structures

Supplementary Figure 1 HPLC traces of the individual steviol glucoside authentic standards and the mixture

Supplementary Figure 2 Metal ion independence of the reaction of UGT76G1

Supplementary Figure 3 Biochemical assays for sugar transfer of STB by UGT76G1

Supplementary Figure 4 Biochemical assays for sugar transfer of Reb A by UGT76G1

Supplementary Figure 5 Biochemical assays for sugar transfer of ST by UGT76G1

Supplementary Figure 6 Biochemical assays for sugar transfer of Reb E by UGT76G1

Supplementary Figure 7 Direct MS of the single sugar transfer reaction at R1 position of the self-made steviol monoglucoside

Supplementary Figure 8 Direct MS of the single sugar transfer reaction at R2 position of the self-made steviol monoglucoside

Supplementary Figure 9 Experimental Fo-Fc difference electron density and the final refined 2Fo-Fc electron density for the bound ligands in the structures

Supplementary Figure 10 Binding mode of UDP in UGT76G1 and UDPG in other UGT enzymes

Supplementary Figure 11 Role of the residue His 25 in the catalysis of UGT76G1

Supplementary Figure 12 Stereo view of the substrate binding pocket of UGT76G1

Supplementary Figure 13 Comparison of the binding orientations for R1 reaction and R2 reaction

Supplementary Figure 14 MS and MS/MS of the reactions of 4-nitrophenyl β-D-glucopyranoside

Supplementary Table 1 The synthetic encoding sequence of UGT76G1 and the primers used in this study

The synthetic UGT76G1 encoding sequence	ATGGAAAATAAAACGGAGACCACCGTTCGCCGGCGCCGGAGAATAATATTATT<br>CCCGGTACCATTTCAAGGCCACATTAACCCAATTCTTCAGCTAGCCAATGTGTTG<br>TACTCTAAAGGATTCAGTATCACCATCTTTCACACCAACTTCAACAAACCCAAAA<br>CATCTAATTACCCTCACTTCACTTTCAGATTCATCCTCGACAACGACCCACAAGA<br>CGAACGCATTTCCAATCTACCGACTCATGGTCCGCTCGCTGGTATGCGGATTCC<br>GATTATCAACGAACACGGAGCTGACGAATTACGACGCGAACTGGAACTGTTGA<br>TGTTAGCTTCTGAAGAAGATGAAGAGGTATCGTGTTTAATCACGGATGCTCTTT<br>GGTACTTCGCGCAATCTGTTGCTGACAGTCTTAACCTCCGACGGCTTGTTTTGAT<br>GACAAGCAGCTTGTTTAATTTTCATGCACATGTTTCACTTCCTCAGTTTGATGAG<br>CTTGGTTACCTCGATCCTGATGACAAAACCCGTTTGGAAGAACAAGCGAGTGG<br>GTTTCCTATGCTAAAAGTGAAAGACATCAAGTCTGCGTATTCGAACTGGCAAAT<br>ACTCAAAGAGATATTAGGGAAGATGATAAAACAAACAAAAGCATCTTCAGGAG<br>TCATCTGGAACTCATTTAAGGAACTCGAAGAGTCTGAGCTCGAAACTGTTATCC<br>GTGAGATCCCGGCTCCAAGTTTCTTGATACCACTCCCCAAGCATTTGACAGCCTC<br>TTCCAGCAGCTTACTAGACCACGATCGAACCGTTTTTCAATGGTTAGACCAACAA<br>CCGCCAAGTTCGGTACTGTATGTTAGTTTTGGTAGTACTAGTGAAGTGGATGAG<br>AAAGATTTCTTGGAAATAGCTCGTGGGTTGGTTGATAGCAAGCAGTCGTTTTTA<br>TGGGTGGTTCGACCTGGGTTTGTCAAGGGTTCGACGTGGGTCGAACCGTTGCC<br>AGATGGGTTCTTGGGTGAAAGAGGACGTATTGTGAAATGGGTTCCACAGCAAG<br>AAGTGCTAGCTCATGGAGCAATAGGCGCATTCTGGACTCATAGCGGATGGAAC<br>TCTACGTTGGAAAGCGTTTGTGAAGGTGTTCCTATGATTTTCTCGGATTTTGGGC<br>TCGATCAACCGTTGAATGCTAGATACATGAGTGATGTTTTGAAGGTAGGGGTGT<br>ATTTGGAAAATGGGTGGGAAAGAGGAGAGATAGCAAATGCAATAAGAAGAGT<br>TATGGTGGATGAAGAAGGAGAATACATTAGACAGAATGCAAGAGTTTTGAAAC<br>AAAAGGCAGATGTTTCTTTGATGAAGGGTGGTTCGTCTTACGAATCATTAGAGT<br>CTCTAGTTTCTTACATTTCATCGTTG
Primers used to Subclone UGT76G1 into pET21a	5’- GGGAATTCCATATGGAAAATAAAACGGAGACCA-3’<br>5’- CCCTCGAGCAACGATGAAATGTAAGAAACTAGAG-3’
Primers used for the mutations	
H25A	5’-CCGGTACCATTTCAAGGCGCCATTAACCCAATTCTTC-3’<br>5’-GAAGAATTGGGTTAATGGCGCCTTGAAATGGTACCGG-3’
H25N	5’- GGTACCATTTCAAGGCAACATTAACCCAATTCTTC-3’<br>5’- GAAGAATTGGGTTAATGTTGCCTTGAAATGGTACCG-3’
D124N	5’-ATCGTGTTTAATCACGAATGCTCTTTGGTACTTCG-3’<br>5’-CGAAGTACCAAAGAGCATTCGTGATTAAACACGAT-3’
D124A	5’-ATCGTGTTTAATCACGGCTGCTCTTTGGTACTTCG-3’<br>5’-CGAAGTACCAAAGAGCAGCCGTGATTAAACACGAT-3’

Supplementary Tables 2 Data collection and refinement statistics of X-ray structures

	UGT76G1 +UDP (6INF)	UGT76G1 H25A +UDP (6ING)	UGT76G1 +UDP+Reb A (6INI)	UGT76G1 +UDP+Rubu(6INH)
**Data collection**				
Space group	$P3_121$	$P3_121$	$P3_121$	$P3_121$
Cell dimensions				
a, *b*, *c* (Å)	99.16, 99.16, 89.04	98.93, 98.93, 89.10	97.66,97.66, 89.63	97.53, 97.53, 90.73
α, β, γ (°)	90, 90, 120	90, 90, 120	90, 90, 120	90, 90, 120
Resolution (Å)	49.58 - 1.69 (1.73 - 1.69) *	85.68 - 1.70 (1.74 - 1.70) *	42.88 - 1.70 (1.74 - 1.70) *	84.46-2.10 (2.15-2.10) *
R_{merge}	0.069 (1.007)	0.086 (3.288)	0.084 (1.139)	0.127 (0.290)
I / σI	20.2 (4.0)	13.2 (2.4)	18.9 (4.6)	7.2 (3.9)
Completeness (%)	100 (100)	100 (100)	100 (100)	100 (99.9)
Redundancy	19.2 (19.4)	9.6 (9.7)	19.1 (19.2)	5.3 (5.3)
CC (1/2)	0.999 (0.948)	0.988 (0.451)	0.999 (0.978)	0.973 (0.916)
**Refinement**				
Resolution (Å)	49.58 - 1.69	85.68 - 1.70	42.88 - 1.70	84.46 - 2.10
No. reflections	56938 (4173)	55785(4095)	54657 (4025)	29548 (2159)
R_{work} / R_{free}	0.178 / 0.207	0.175 / 0.204	0.161 / 0.190	0.167 / 0.198
No. atoms	3900	3920	4049	3987
Protein	3683	3620	3655	3663
Ligand/ion	25	43	121	116
Water	192	257	273	199
B-factors	38.27	45.69	33.79	25.79
Protein	38.54	45.73	33.54	24.51
Ligand/ion	23.19	43.46	33.78	45.47
Water	35.04	45.44	37.22	37.96
R.m.s. deviations				
Bond lengths (Å)	0.011	0.009	0.008	0.009
Bond angles (°)	1.46	1.18	1.63	1.31

*One crystal was used for data collection and structure determination. The resolution limits were determined by half-dataset correlation (CC (1/2)) and values in parentheses are for highest-resolution shell.

Supplementary Figure 1

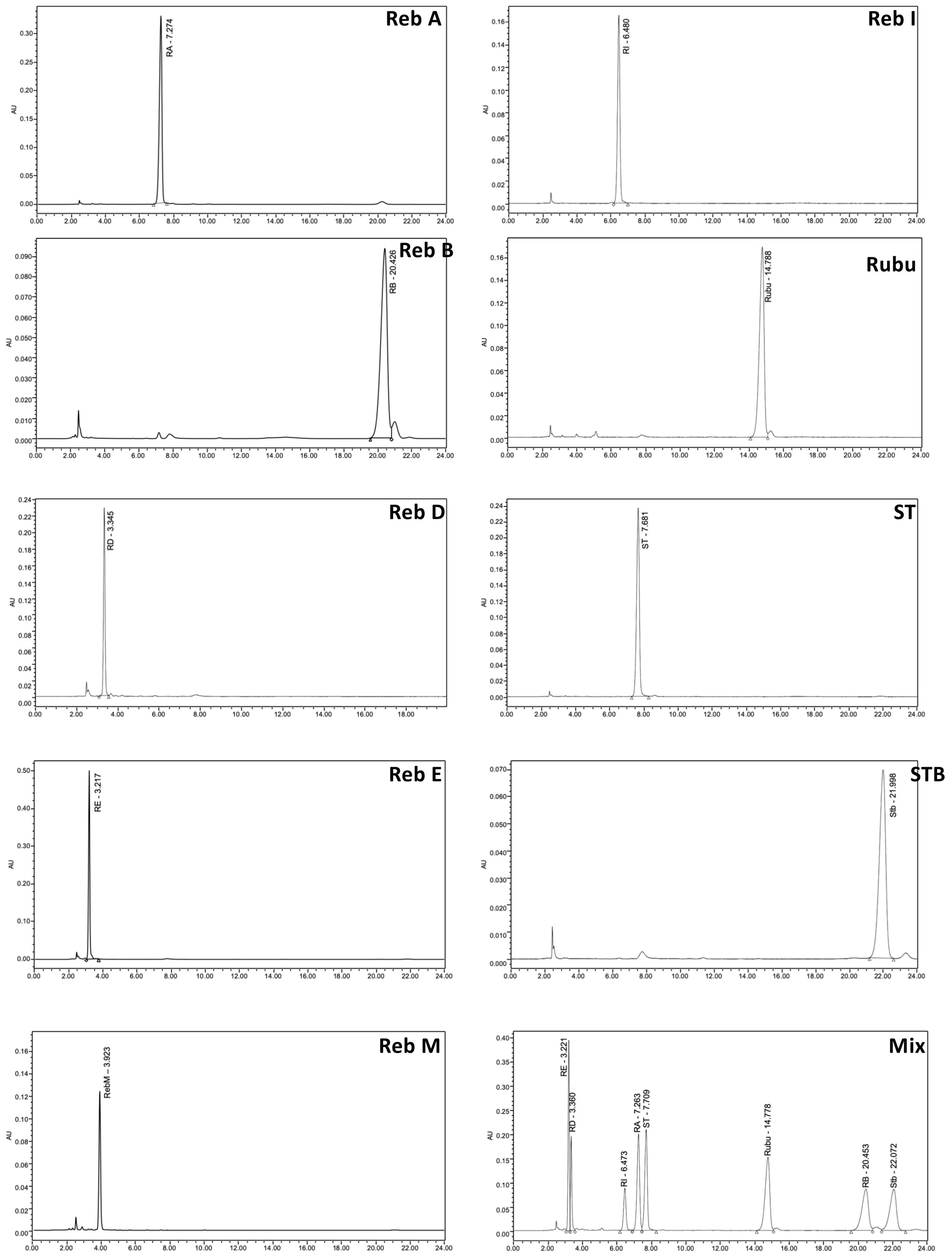

Supplementary Figure 1 HPLC traces of the individual steviol glucoside authentic standards and the mixture. The retention time of the individual one is identical to which shown in the mixture of the authentic standards.

Supplementary Figure 2

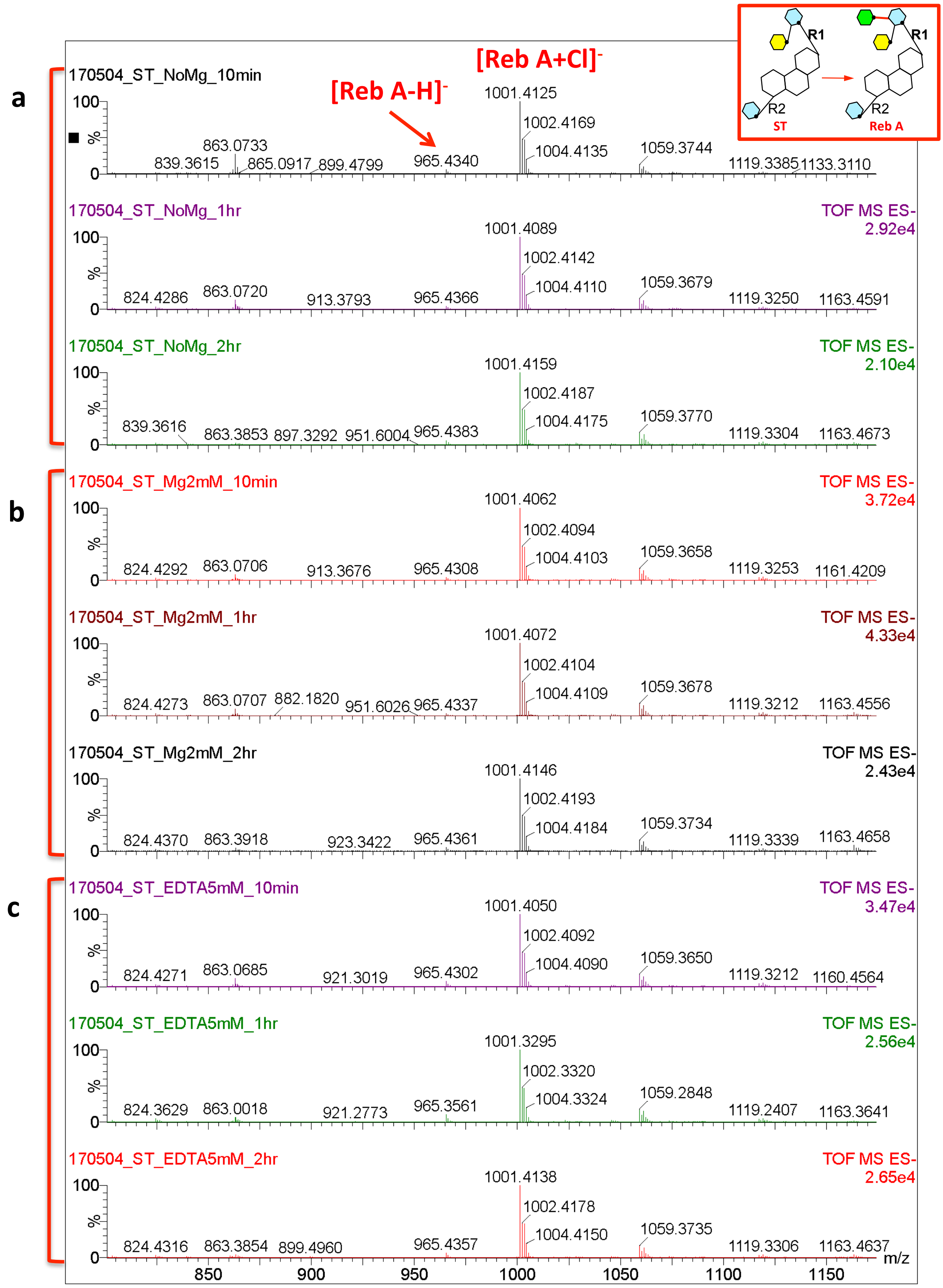

Supplementary Figure 2 Metal ion independence of the reaction of UGT76G1. Direct MS of the reactions of UGT76G1 in the absence of Mg^{2+}, with supplemental Mg^{2+} or the chelating

EDTA. The product yields have not significantly changed upon the presence of Mg^{2+} or the chelating reagents

a) MS of the reaction of ST by 0.15 mg ml^{-1} UGT76G1 without any supplemental Mg^{2+}, which were sampled at the time course of 10 min, 1 hour and 2 hours. The two main negative ions derived from the product Reb A are labelled. The reaction of ST catalysed by UGT76G1 is shown in the red box during the course of the assay, when the R2 reaction has not started significantly.
b) MS of the reaction of ST by 0.15 mg ml^{-1} UGT76G1 with 2 mM Mg^{2+}, which were sampled at the time course of 10 min, 1 hour and 2 hours.
c) MS of the reaction of ST by 0.15 mg ml^{-1} UGT76G1 with the addition of 5 mM EDTA to remove any residual free Mg^{2+}, which were sampled at the time course of 10 min, 1 hour and 2 hours.

Supplementary Figure 3

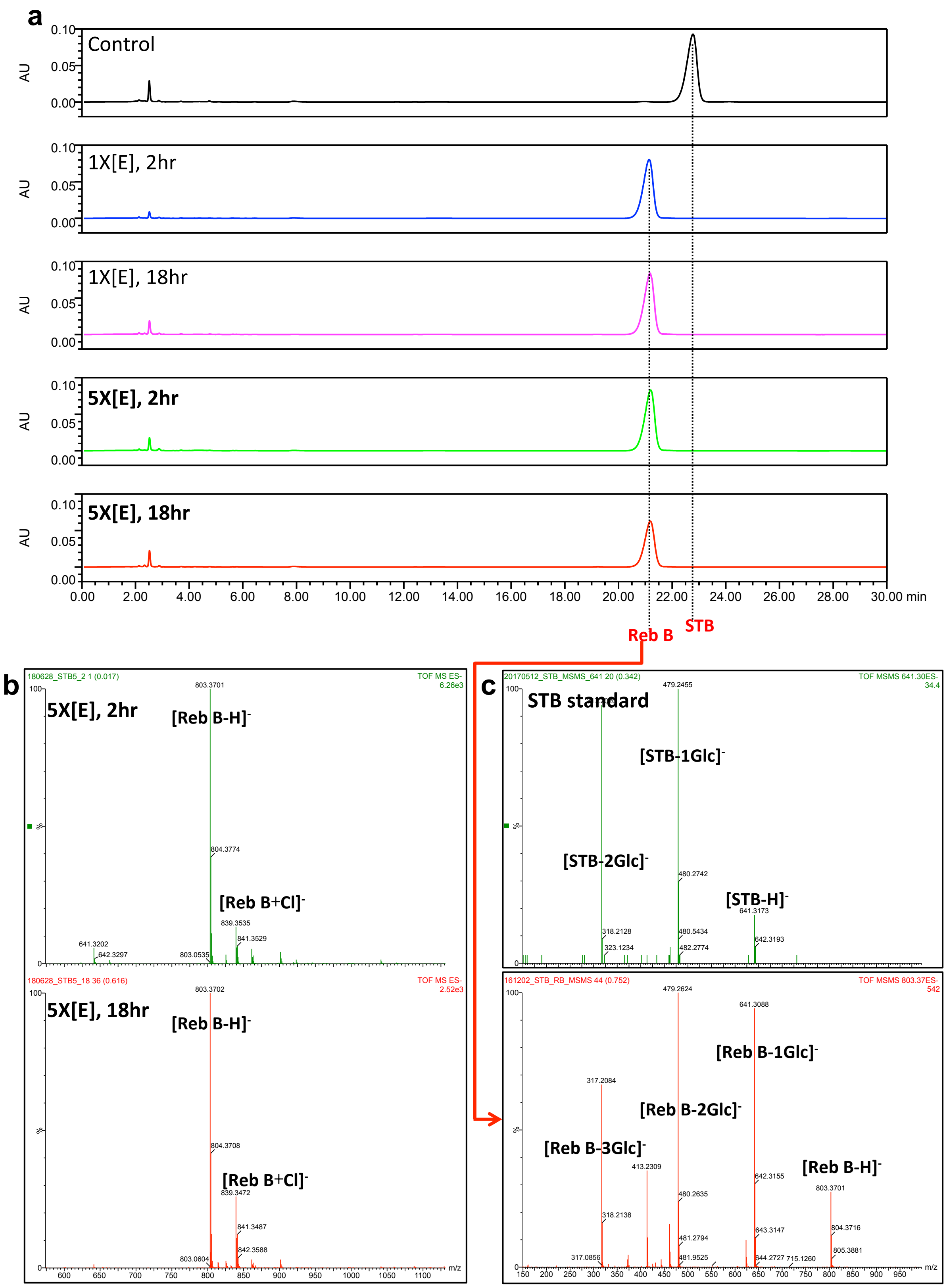

Supplementary Figure 3 Biochemical assays for sugar transfer of STB by UGT76G1

(a) HPLC traces of the reactions of STB. The five HPLC traces from top to bottom represent the following reaction conditions: no enzyme for 18 hours (black), 0.03 mg ml^{-1} enzyme (1x) for 2 hours (blue), 0.03 mg ml^{-1} enzyme (1x) for 18 hours (purple), 0.15 mg ml^{-1} enzyme (5x) for 2 hours (green) and 0.15 mg ml^{-1} enzyme (5x) for 18 hours (red). The yields of the products are related to the enzyme concentration and the reaction duration. The product is identified to be Reb B by the authentic standard.

(b) Direct MS of two reactions of STB by 0.15 mg ml^{-1} UGT76G1 (5x) for 2 hours and 18 hours. The two main negative ions derived from the product Reb B are labelled and show the same characteristics in terms of the relative contents of the product as a function of the reaction duration as shown by HPLC.

(c) MS/MS of the authentic STB standard and the collected HPLC peak of the product Reb B. The negative ion of $[STB\text{-}H]^-$ with m/z at 641.3, or $[Reb\ B\text{-}H]^-$ with m/z at 803.4 was specifically isolated and characterized. In MS/MS, there are not any labile ester bonds in the samples, so that they showed the different fragmentation profile from those steviol glucosides containing ester bond at R2 position.

Supplementary Figure 4

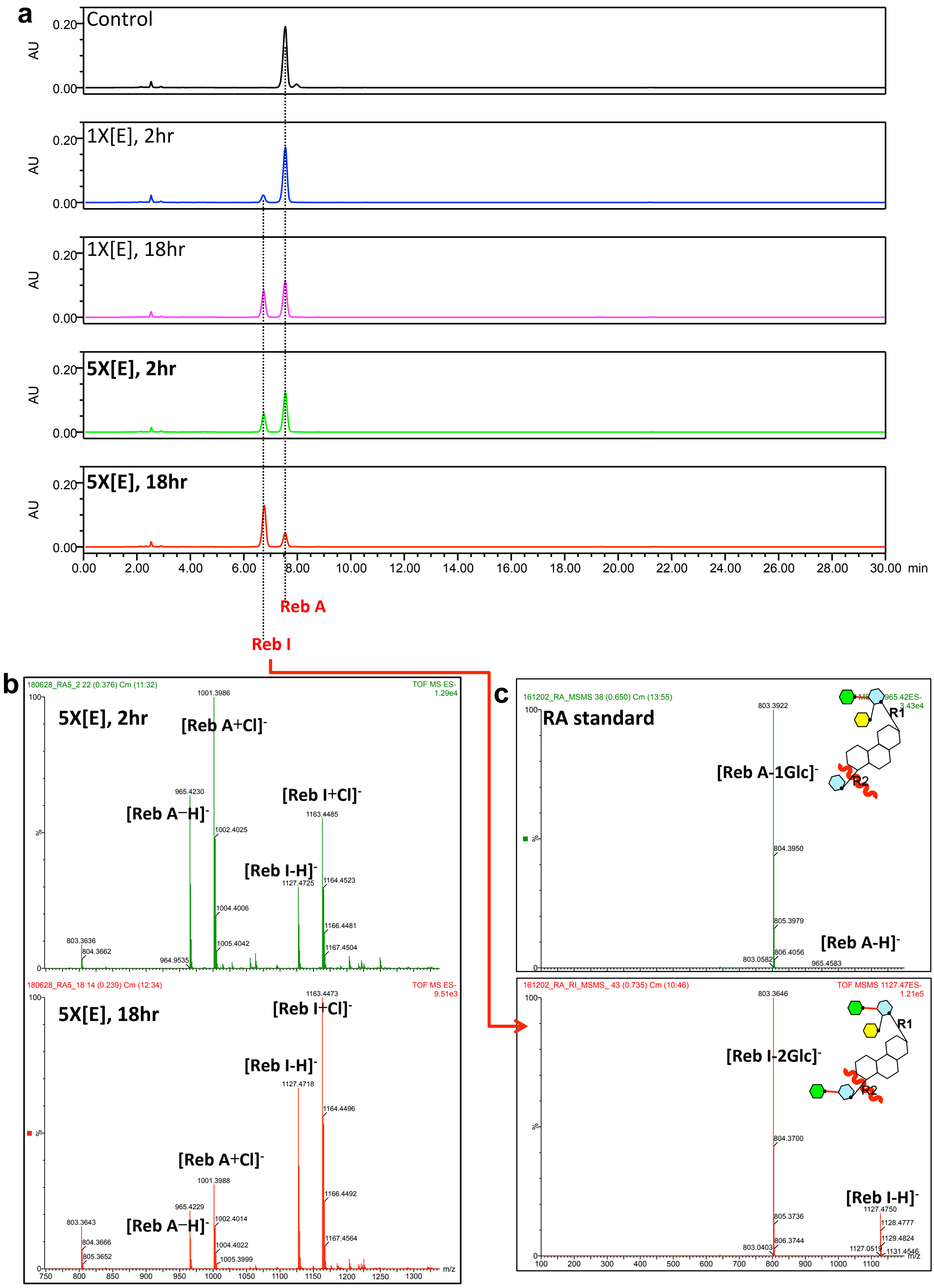

Supplementary Figure 4 Biochemical assays for sugar transfer of Reb A by UGT76G1

(a) HPLC traces of the reactions of Reb A. The five HPLC traces from top to bottom represent the following reaction conditions: no enzyme for 18 hours (black), 0.03 mg ml^{-1} enzyme (1x) for 2 hours (blue), 0.03 mg ml^{-1} enzyme (1x) for 18 hours (purple), 0.15 mg ml^{-1} (5x) of the enzyme for 2 hours (green) and 0.15 mg ml^{-1} (5x) of the enzyme for 18 hours (red). The substrate Reb A has not been used up during the assay. The substrate consumption (Reb A) and the product yields are related to the enzyme concentration and the reaction duration. The product is identified to be Reb I by the authentic standard.

(b) Direct MS of two reactions of Reb A by 0.15 mg ml^{-1} UGT76G1 (5x) for 2 hours and 18 hours. The two main negative ions derived from the substrate Reb A and the product Reb I are labelled and show the same characteristics in terms of the relative contents of the product as a function of the reaction duration as shown by HPLC.

(c) MS/MS of the authentic Reb A standard and the collected HPLC peak of the product Reb I. The negative ion of $[\text{Reb A-H}]^-$ with m/z at 965.4 or $[\text{Reb I-H}]^-$ with m/z at 1127.5 was specifically isolated and characterized. In MS/MS, the most labile ester bond breaks first, which was consistently indicated by the abundant fragment ions of Reb A and Reb I. The inlet suggests where the ester bond breaks first in MS/MS fragmentation.

Supplementary Figure 5

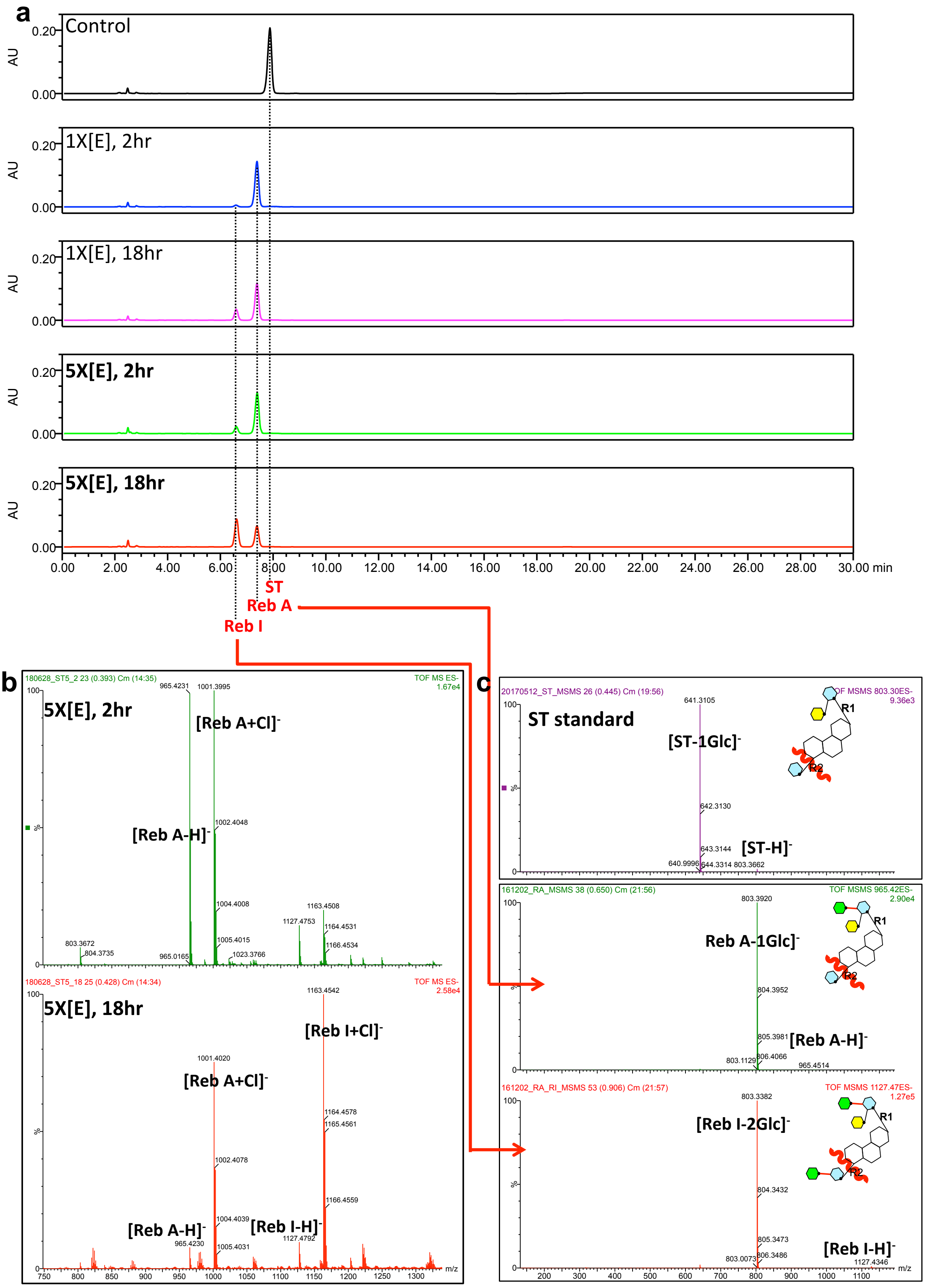

Supplementary Figure 5 Biochemical assays for sugar transfer of ST by UGT76G1

(a) HPLC traces of the reactions of ST. The five HPLC traces from top to bottom represent the following reaction conditions: no enzyme for 18 hours (black), 0.03 mg ml^{-1} enzyme (1x) for 2 hours (blue), 0.03 mg ml^{-1} enzyme (1x) for 18 hours (purple), 0.15 mg ml^{-1} enzyme (5x) for 2 hours (green) and 0.15 mg ml^{-1} enzyme (5x) for 18 hours (red). The substrate ST has been used up for each assay condition. The yields of the two successive products showed the reaction order and are related to the enzyme concentration and the reaction duration. Two products were identified to be Reb A and Reb I by the authentic standards.

(b) Direct MS of two reactions of ST by 0.15 mg ml^{-1} UGT76G1 (5x) for 2 hours and 18 hours. The two main negative ions derived from two products Reb A and Reb I are labelled and show the same characteristics in terms of the relative contents of the product as a function of the reaction duration as shown by HPLC.

(c) MS/MS of the authentic ST standard and the collected HPLC peaks of the product Reb A and Reb I. The negative ion of $[ST\text{-}H]^-$ with m/z at 803.3, $[Reb\ A\text{-}H]^-$ with m/z at 965.4 or $[Reb\ I\text{-}H]^-$ with m/z at 1127.5 was specifically isolated and characterized. In MS/MS, the most labile ester bond breaks first, which was consistently indicated by the abundant fragment ions of ST, Reb A and Reb I. The inlet suggests where the ester bond breaks first in MS/MS fragmentation.

Supplementary Figure 6

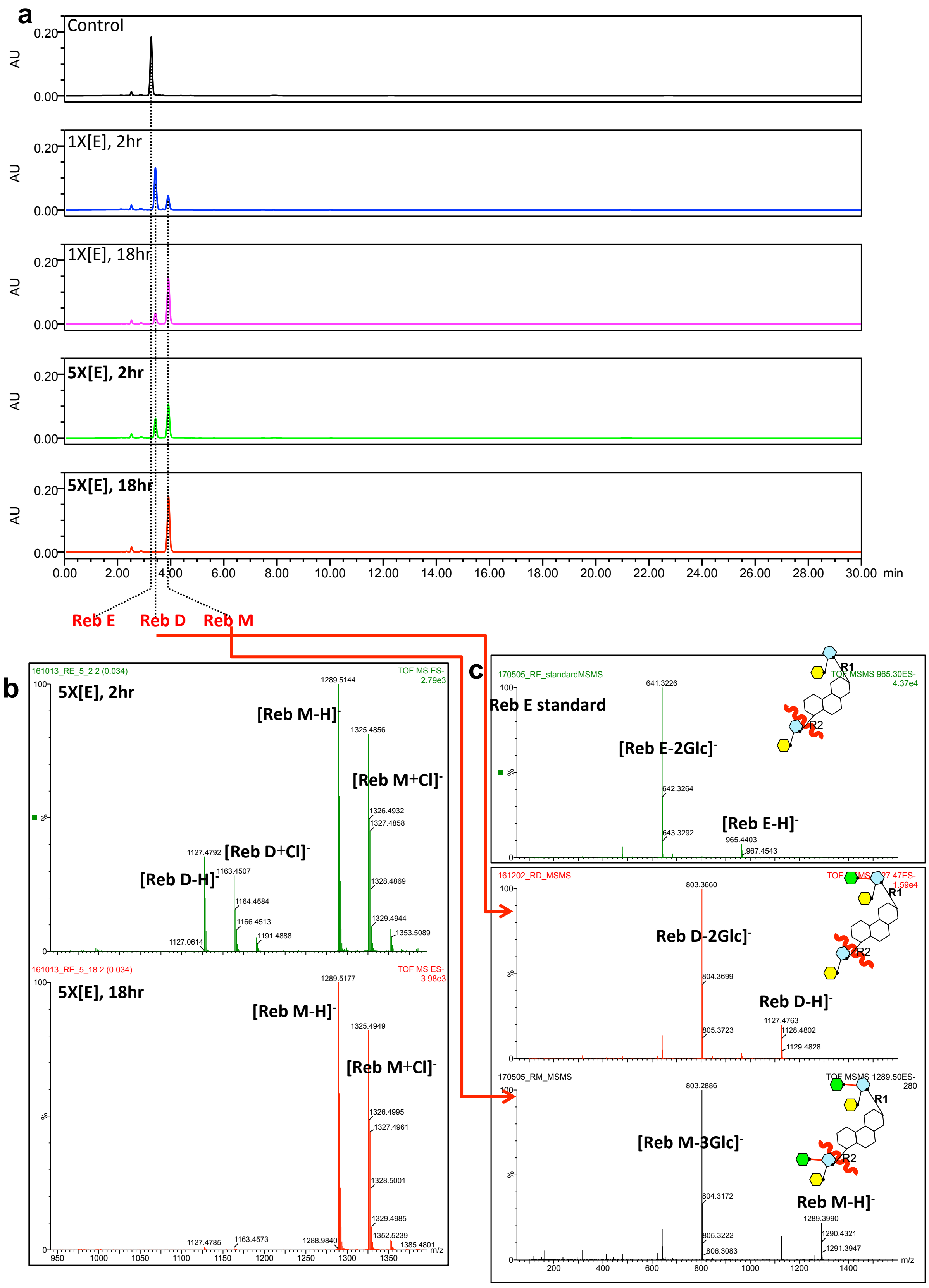

Supplementary Figure 6 Biochemical assays for sugar transfer of Reb E by UGT76G1

(a) HPLC traces of the reactions of Reb E. The five HPLC traces from top to bottom represent the following reaction conditions: no enzyme for 18 hours (black), 0.03 mg ml^{-1} enzyme (1x) for 2 hours (blue) and 18 hours (purple), 0.15 mg ml^{-1} enzyme (5x) for 2 hours (green) and 18 hours (red). The substrate Reb E has been used up for each assay condition. The yields of the two successive products showed the reaction order and are related to the enzyme concentration and the reaction duration. Two products were identified to be Reb D and Reb M by the authentic standards.

(b) Direct MS of two reactions of Reb E by 0.15 mg ml^{-1} of UGT76G1 (5x) for 2 hours and 18 hours. The two main negative ions derived from two products Reb D and Reb M are labelled and show the same characteristics in terms of the relative contents of the product as a function of the reaction duration as shown by HPLC.

(c) MS/MS of the authentic Reb E standard and the collected HPLC peaks of the product Reb D and Reb M. The negative ion of $[\text{Reb E-H}]^-$ with m/z at 965.4, $[\text{Reb D-H}]^-$ with m/z at 1127.5 or $[\text{Reb M-H}]^-$ with m/z at 1289.4 was specifically isolated and characterized. In MS/MS, the most labile ester bond breaks first, which was consistently indicated by the abundant fragment ions of Reb E, Reb D and Reb M. The inlet suggests where the ester bond breaks first in MS/MS fragmentation.

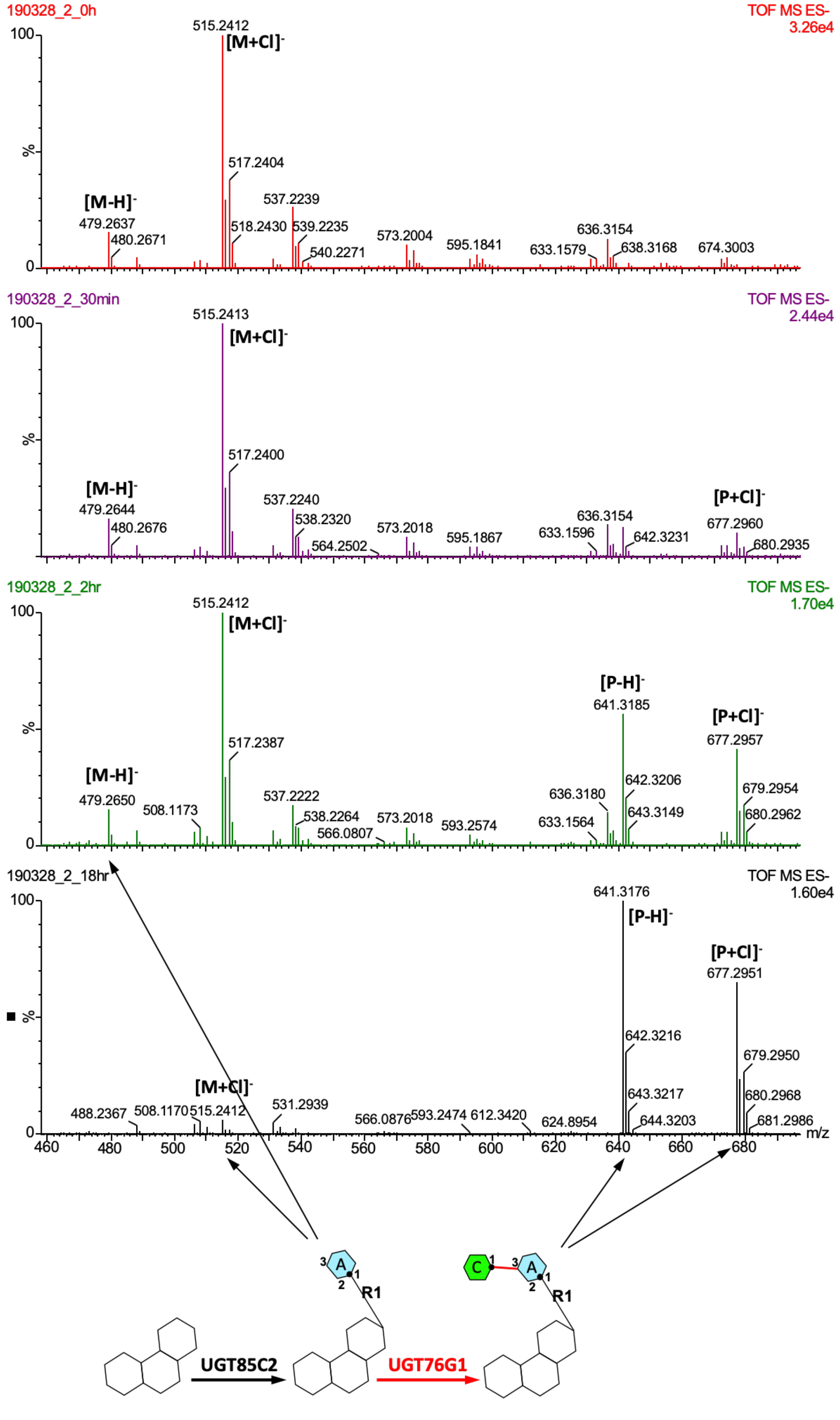
190328_2_0h
TOF MS ES-
3.26e4
515.2412
[M+Cl]-
[M-H]-
479.2637
480.2671
517.2404
518.2430
537.2239
539.2235
540.2271
573.2004
595.1841
633.1579
636.3154
638.3168
674.3003
190328_2_30min
TOF MS ES-
2.44e4
515.2413
[M+Cl]-
[M-H]-
479.2644
480.2676
517.2400
537.2240
538.2320
564.2502
573.2018
595.1867
633.1596
636.3154
642.3231
[P+Cl]-
677.2960
680.2935
190328_2_2hr
TOF MS ES-
1.70e4
515.2412
[M+Cl]-
[M-H]-
479.2650
508.1173
517.2387
537.2222
538.2264
566.0807
573.2018
593.2574
633.1564
636.3180
[P-H]-
641.3185
642.3206
643.3149
[P+Cl]-
677.2957
679.2954
680.2962
190328_2_18hr
TOF MS ES-
1.60e4
641.3176
[P-H]-
[P+Cl]-
677.2951
642.3216
643.3217
644.3203
679.2950
680.2968
681.2986
[M+Cl]-
488.2367
508.1170
515.2412
531.2939
566.0876
593.2474
612.3420
624.8954
m/z
%
UGT85C2
UGT76G1
R1
A
C

Supplementary Figure 7 Direct MS of the single sugar transfer reaction at R1 position of the self-made steviol monoglucoside. The reaction was catalyzed by 0.03 mg ml^{-1} UGT76G1 (1x) and the aliquots (from top to bottom) were analyzed at 0, 0.5, 2 and 18 hours. The main negative ions derived from the products are labeled and related to the chemical compounds in the reaction scheme. The consumption of the substrate and the yields of the products are related to the reaction duration.

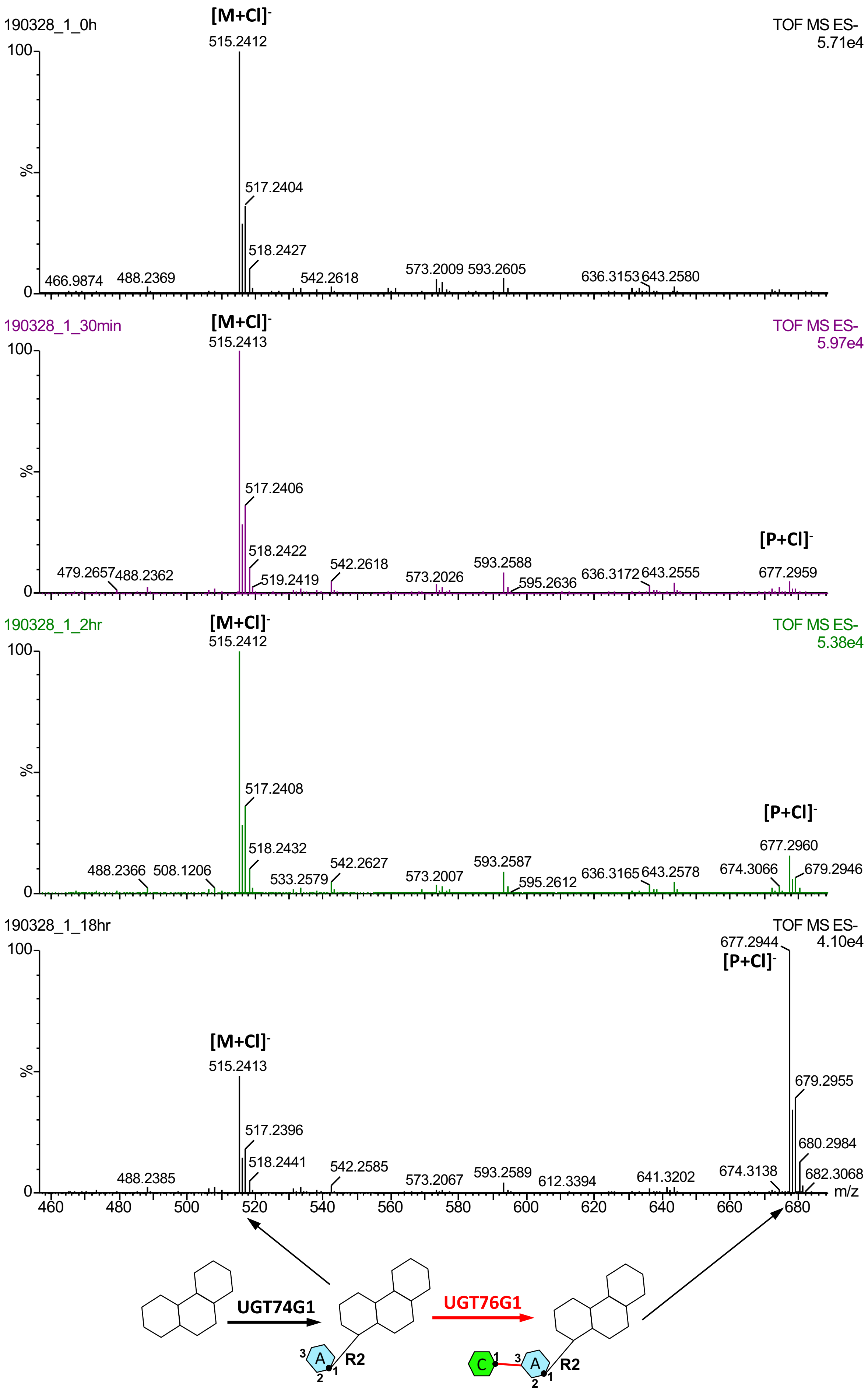

190328_1_0h
TOF MS ES-
5.71e4
[M+Cl]-
515.2412
517.2404
518.2427
466.9874
488.2369
542.2618
573.2009
593.2605
636.3153
643.2580
190328_1_30min
TOF MS ES-
5.97e4
[M+Cl]-
515.2413
517.2406
518.2422
519.2419
479.2657
488.2362
542.2618
573.2026
593.2588
595.2636
636.3172
643.2555
[P+Cl]-
677.2959
190328_1_2hr
TOF MS ES-
5.38e4
[M+Cl]-
515.2412
517.2408
518.2432
488.2366
508.1206
533.2579
542.2627
573.2007
593.2587
595.2612
636.3165
643.2578
674.3066
[P+Cl]-
677.2960
679.2946
190328_1_18hr
TOF MS ES-
4.10e4
677.2944
[P+Cl]-
[M+Cl]-
515.2413
517.2396
518.2441
488.2385
542.2585
573.2067
593.2589
612.3394
641.3202
674.3138
679.2955
680.2984
682.3068
%
m/z
UGT74G1
UGT76G1
R2
A
C

Supplementary Figure 8 Direct MS of the single sugar transfer reaction at R2 position of the self-made steviol monoglucoside. The reaction was catalyzed by 0.03 mg ml^{-1} UGT76G1 (1x) and the aliquots (from top to bottom) were analyzed at 0, 0.5, 2 and 18 hours. The main negative ions derived from the products are labeled and related to the chemical compounds in the reaction scheme. The consumption of the substrate and the yields of the products are related to the reaction duration.

Supplementary Figure 9

a

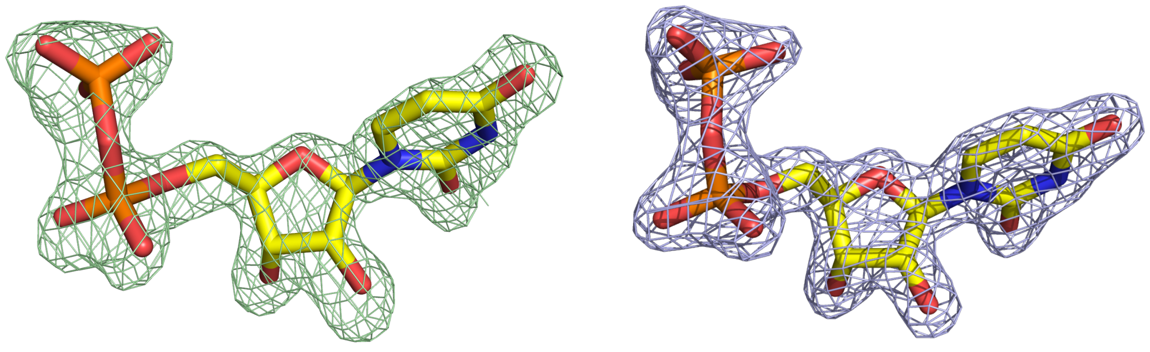

b

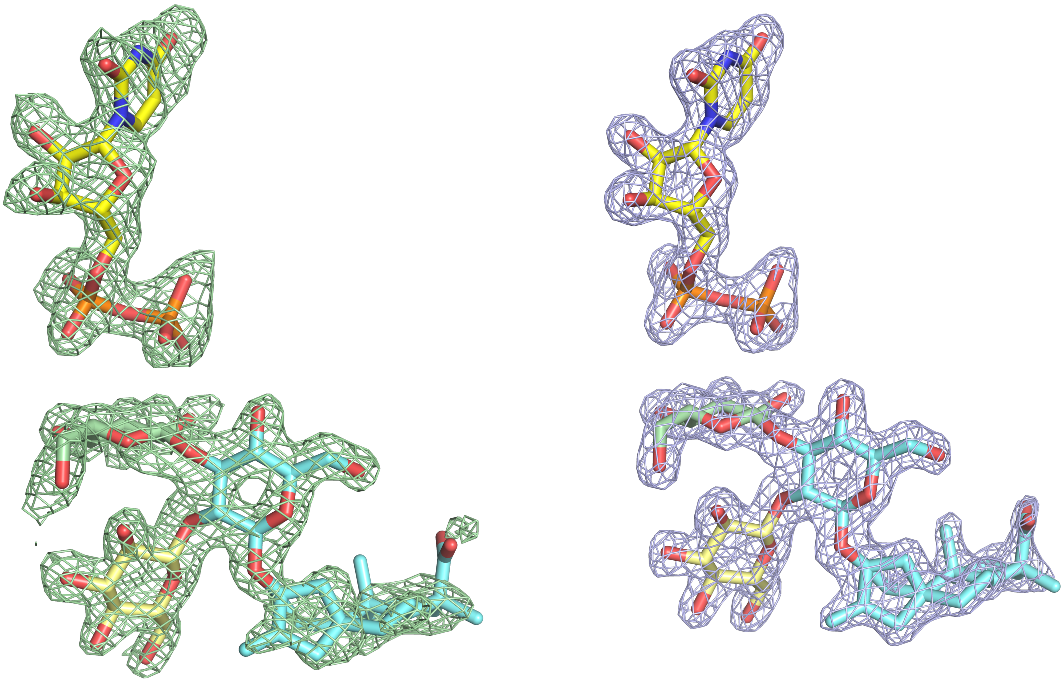

c

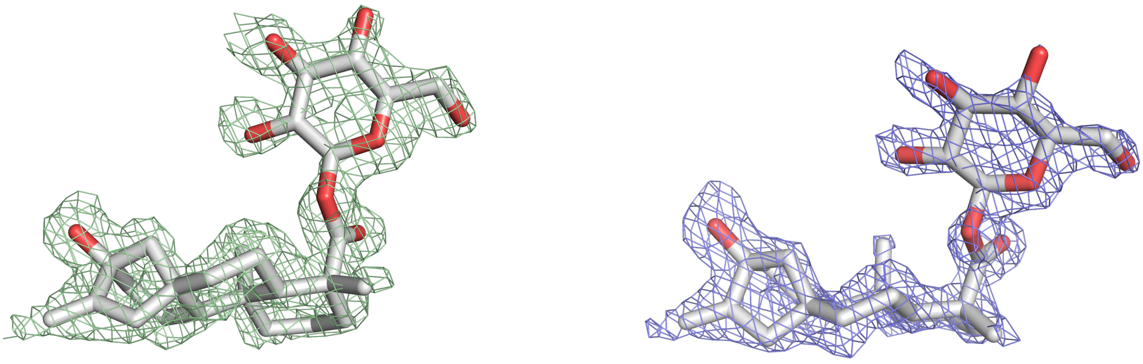

d

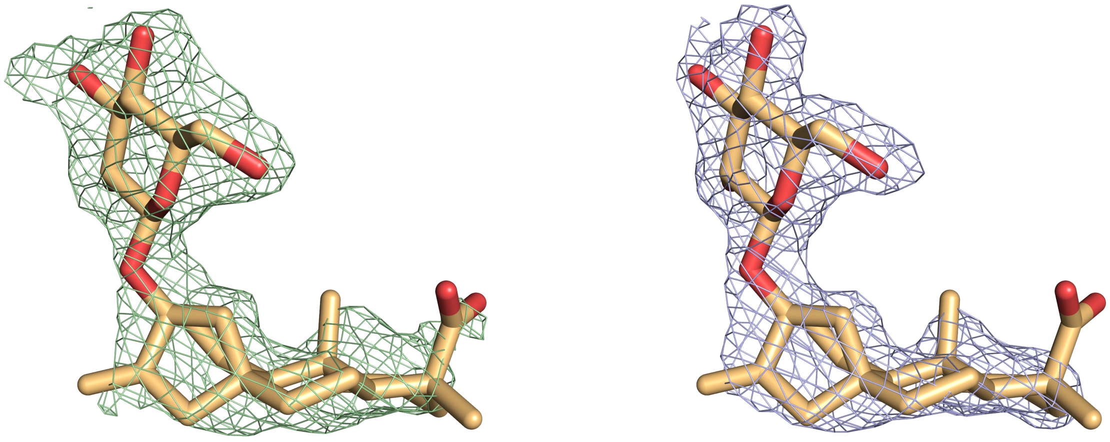

e

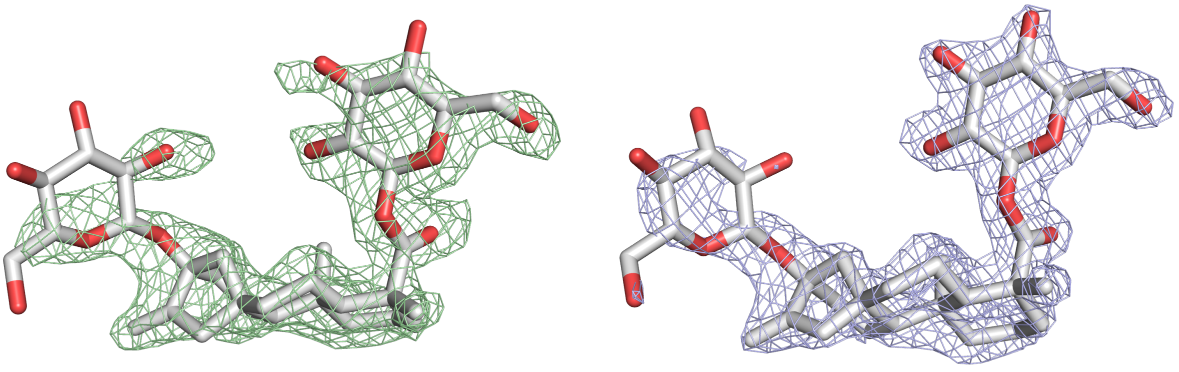

Supplementary Figure 9 Experimental Fo-Fc difference electron density contoured at 3σ in green and the final refined 2Fo-Fc electron density (1σ) in blue for the bound ligands in the structures. The final refined positions of the ligands are shown in stick with oxygens red, nitrogens blue and carbons colored differently for every ligand.

(a) UDP in the binary complex of UGT76G1 and UDP, with carbons colored yellow.

(b) UDP and the reactive Reb A in the ternary complex of UGT76G1/UDP/Reb A, with the same color scheme as shown in Figure 3.

(c) The nonreactive Reb A in the ternary complex of UGT76G1/UDP/Reb A, with carbons colored white.

(d) The reactive Rubu in the ternary complex of UGT76G1/UDP/Rubu, with the same color scheme as shown in Figure 3.

(e) The nonreactive Rubu in the ternary complex of UGT76G1/UDP/Rubu, with carbons colored white.

Supplementary Figure 10

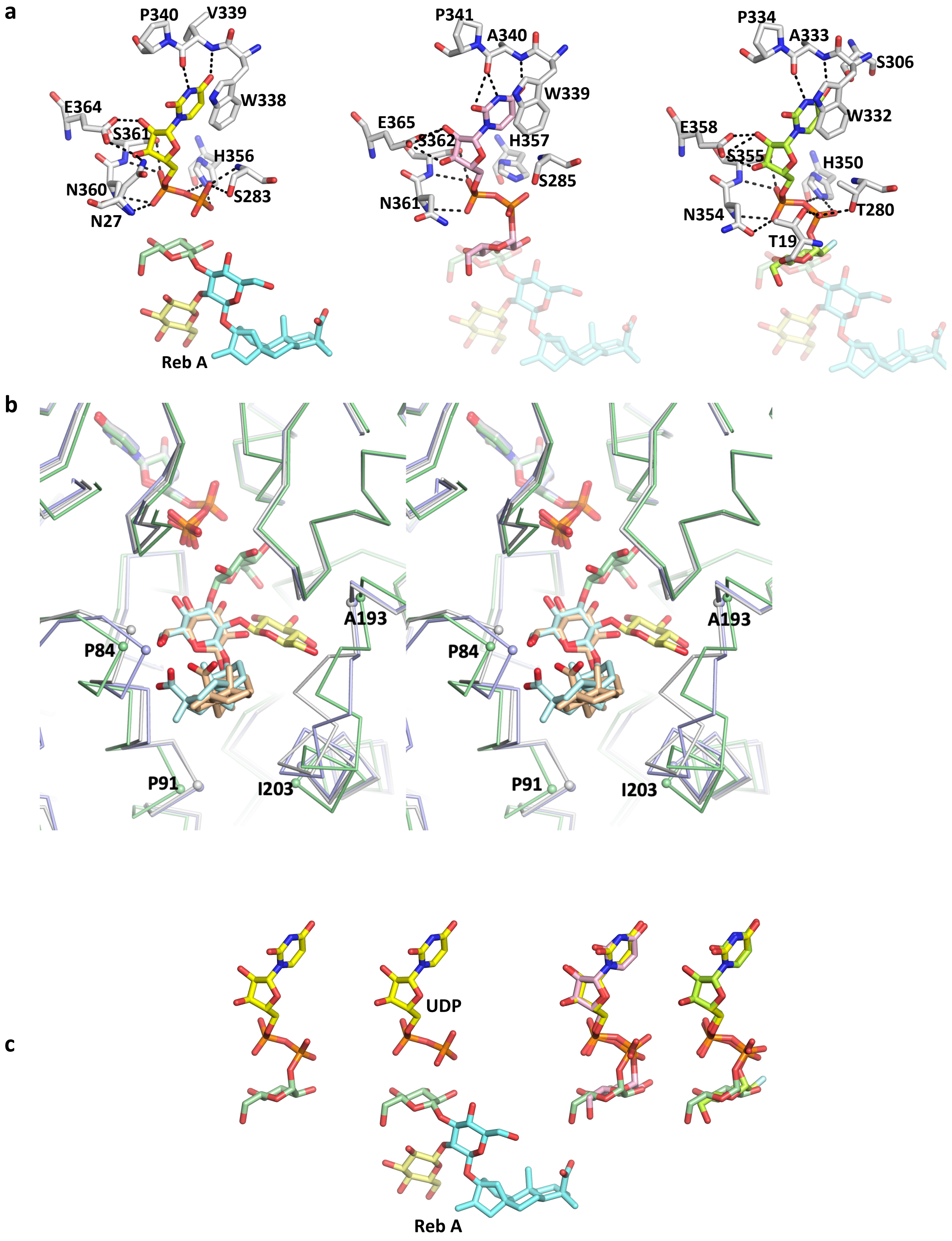

Supplementary Figure 10 Binding mode of UDP in UGT76G1 and UDPG in other UGT enzymes

(a) Comparison of the binding of UDP and UDPG. From the left to right, the figures are showing the similar interactions associated with UDP (with carbons colored yellow) in the ternary complex structure of UGT76G1/UDP/Reb A and UDPG or the analogue in two structure homologs in 2acw (with carbons colored pink) and in 2c1z (with carbons green).

Reb A is also shown transparently in two representations of 2acw and 2c1z, which displays the overlapping of the incoming glucose of Reb A and the glucose of UDPG.

(b) Shifts of UDP and main chain upon Reb A or Rubu binding. The binary structure of the enzyme with UDP is colored with carbons green, oxygens red and nitrogens blue. The ternary complex UGT76G1/UDP/Reb A is colored with carbons white, oxygens red and nitrogens blue, while Reb A is in the same scheme as in Figure 3. The ternary complex UGT76G1/UDP/Rubu is colored with carbons light blue, oxygens red and nitrogens blue, while Rubu is in the same scheme as in Figure 3. The main chains from Pro 84 to Pro 91 and from Ala 193 to Ile 203 are shifted upon the steviol compounds, which are labelled with a ball to show the moving ranges.

(c) Binding model of UDPG of UGT76G1. From the left to right, the figures are showing the binding model of UDPG of UGT76G1; the relative positions to UDP/RebA (with the same color scheme as Figure 3) in the ternary complex structure of UGT76G1/UDP/Reb A; the overlapping comparison of UDPG model of UGT76G1 to the binding mode of UDPG or the analogue in two crystal structure homologs 2acw (with carbons colored pink) and 2c1z (with carbons green).

Supplementary Figure 11

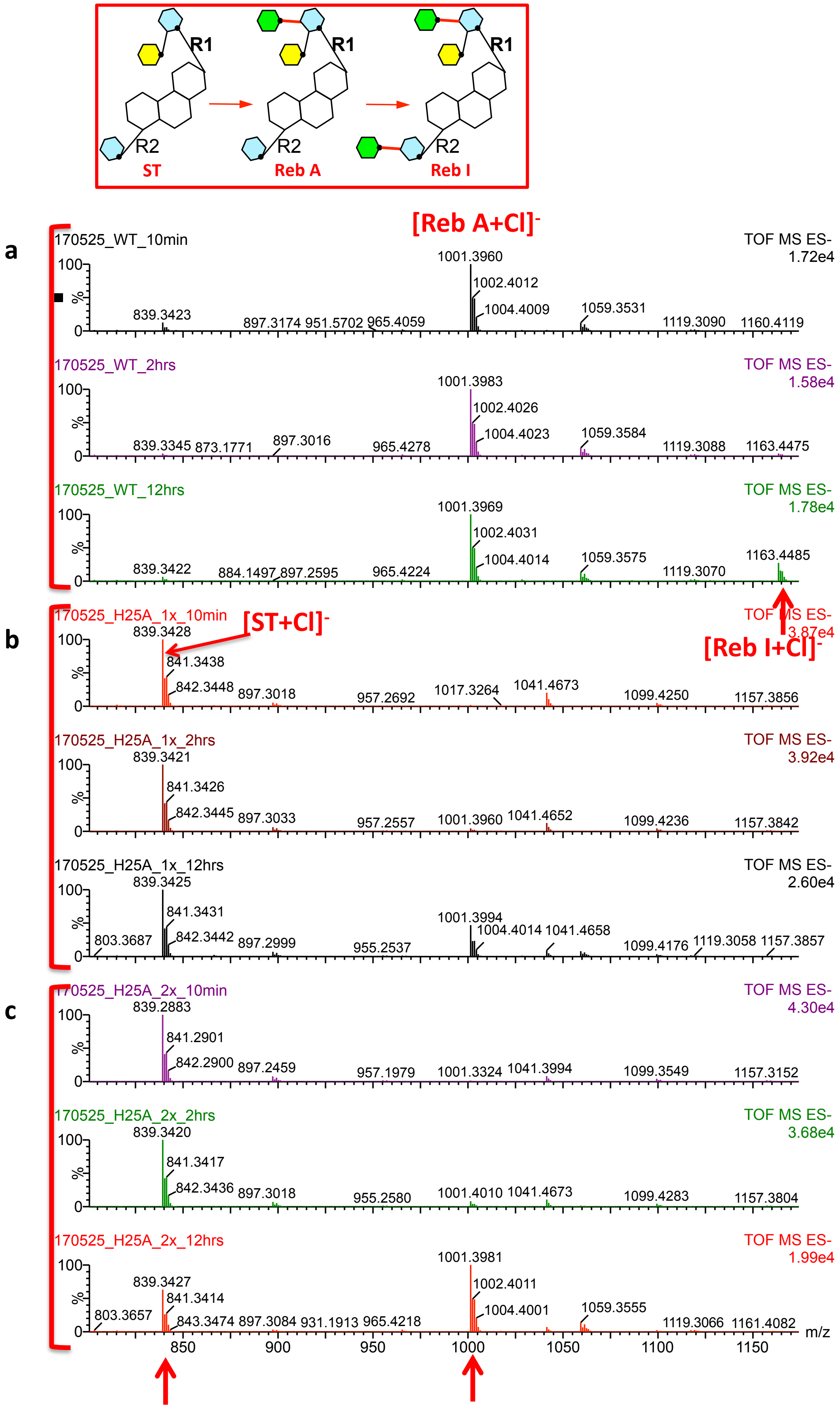

d

	R1 acceptor ST	
	**WT UGT76G1**	**H25A**
**K_m (μM)**	25.5±3.3	194.7±50.0
**k_{cat} (min^{-1})**	41.8±1.4	0.0005±0.00007
**k_{cat}/K_m (S^{-1} mM^{-1})**	27.3	0.00004

e

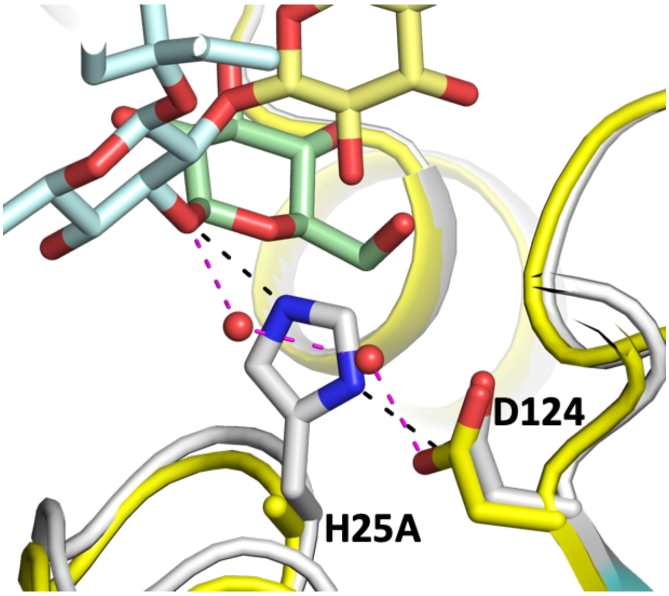

Supplementary Figure 11 Role of the residue His 25 in the catalysis of UGT76G1

(a)-(c) Direct MS of the reactions of UGT76G1 wild type and H25A mutant when ST as the substrate. The main negative ions derived from substrate ST, products Reb A and Reb I are labelled, which show the consumption of substrate and product yield is related to the enzyme concentration and the reaction duration. The reaction scheme of ST catalysed by UGT76G1 is shown in the box.

(a) Reaction of ST by 0.1 mg ml^{-1} wild type UGT76G1. The sample aliquots at the time course of 10 min, 2 hour and 12 hours were measured. The substrate ST has been almost consumed in 10 min and the R2 reaction is much slower and roughly 20% Reb A was converted to Reb I after 12 hours.

(b) Reaction of ST by 0.1 mg ml^{-1} H25A mutant. The sample aliquots at the time course of 10 min, 2 hour and 12 hours were measured. The substrate ST has not been used up even after the reaction of 12 hours while the wild type finishes the R1 reaction in less than 10 min. The yield of Reb A (product of the first R1 reaction) is about half of the remaining substrate ST, suggesting ~30% transformation for 12 hours.

(c) Reaction of ST by 0.2 mg ml^{-1} H25A mutant (2x). The sample at the time course of 10 min, 2 hour and 12 hours were measured. After 12 hours, the remaining substrate ST is about 60% of the produced Reb A, suggesting a 60% turnover (2x).
(d) Kinetic parameters of the wild type and H25A mutant of UGT76G1 in the reaction of ST by UDP quantification assay.
(e) Comparison of the wild type ternary complex structure with Reb A and the H25A mutant complex with UDP. The wild type and H25A structure are shown in cartoon representation with the proteins in white (wild type) and yellow (H25A). The relevant hydrogen bonds to His 25 are shown in dash lines. Two water molecules in H25A structure occupy the position of His 25 and form hydrogen bonds to Asp 124, which act as a similar but much worse proton relay as wild type and retain a very weak activity.

a

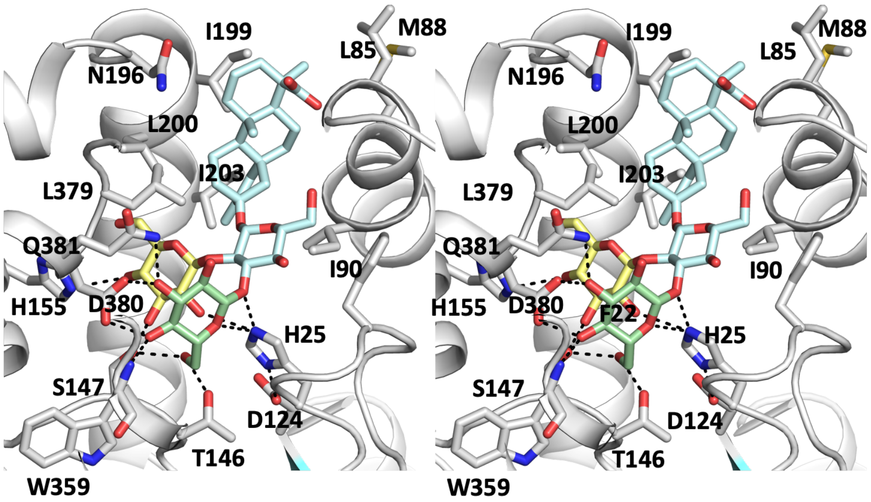

b

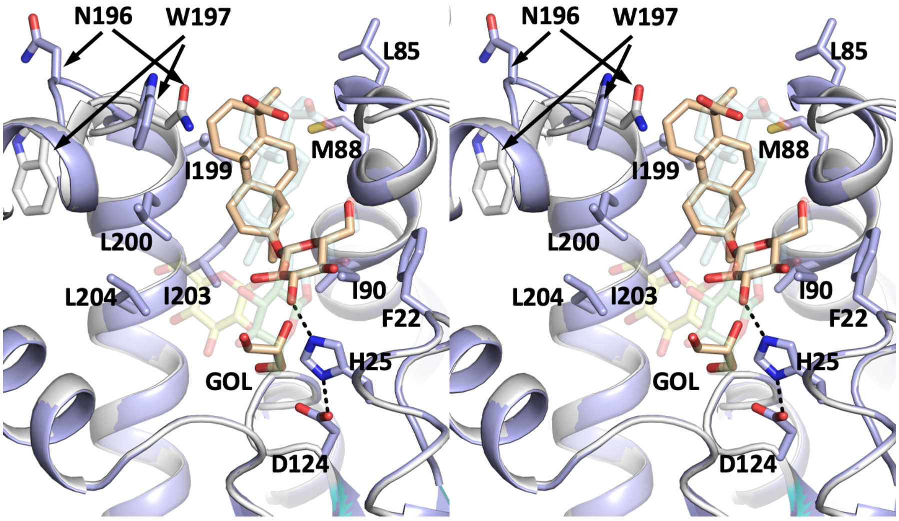

Supplementary Figure 12 Stereo view of the substrate binding pocket of UGT76G1. The structures of complex with Reb A (a) and Rubu (b) are shown in the same representation and color scheme as shown in Figure 3 of the main text.

Supplementary Figure 13

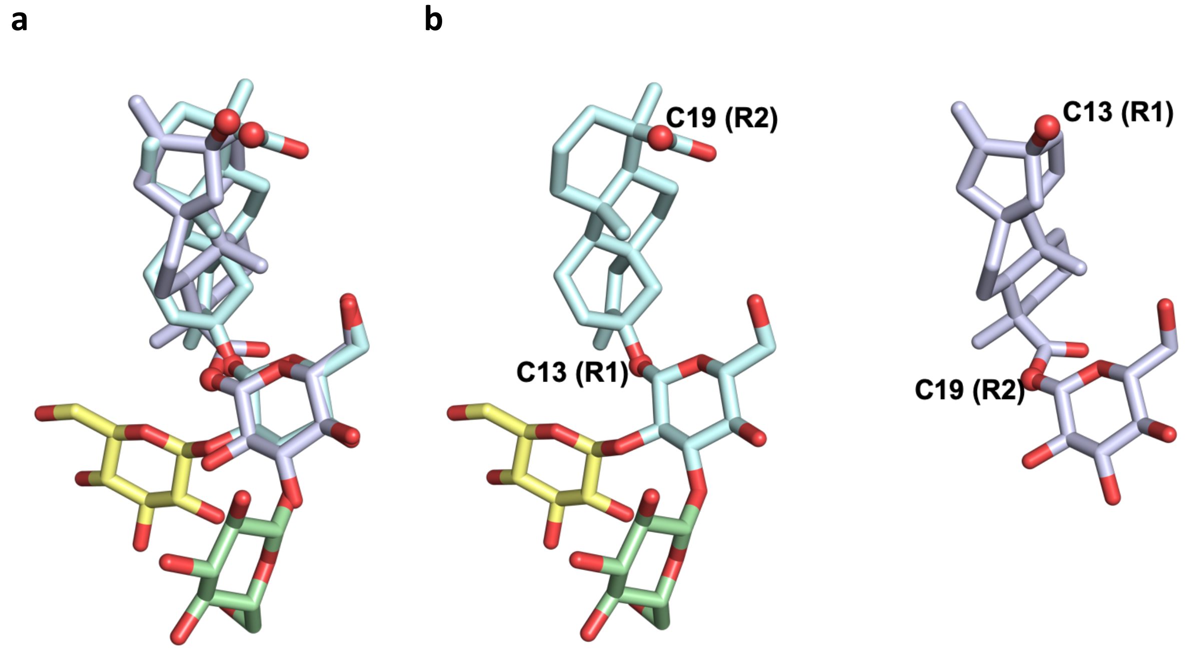

Supplementary Figure 13 Comparison of the binding orientations for R1 reaction and R2 reaction.

(a) Overlapping of Reb A (steviol carbons colored in cyan) in the normal binding mode and the modelled Reb A fragment (steviol carbons colored in light purple) in the flipped mode. The oxygens of C13-hydroxyl (R1) and C19-carboxylate (R2) are shown in red balls, which are almost overlapped between the normal and flipped orientations.

(b) Separation of Reb A (steviol carbons colored in cyan) in the normal binding mode and the modelled Reb A fragment (steviol carbons colored in light purple) in the flipped mode. The positions of C13-hydroxyl (R1) and C19-carboxylate (R2) are labeled individually.

Supplementary Figure 14

a

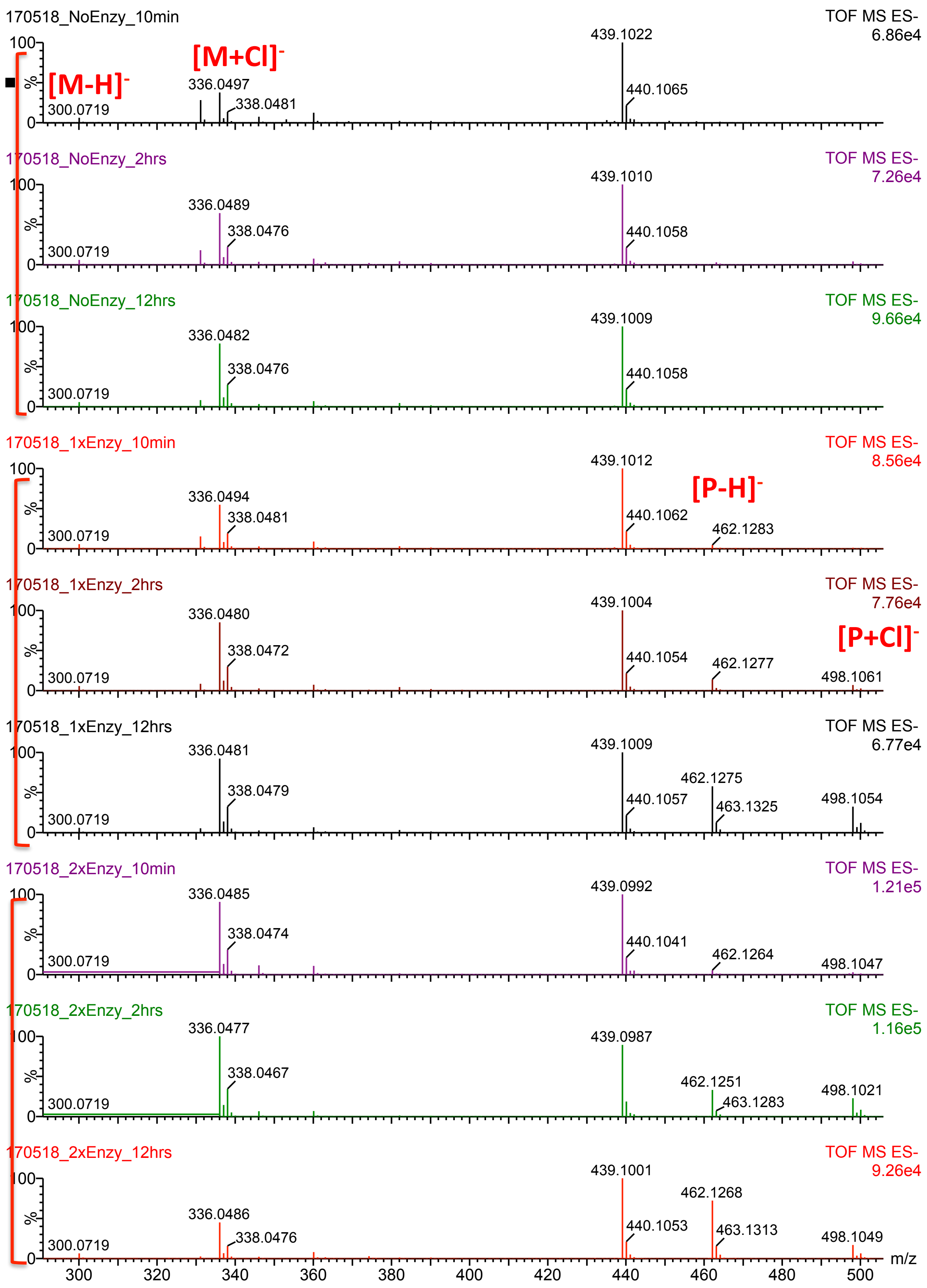

b

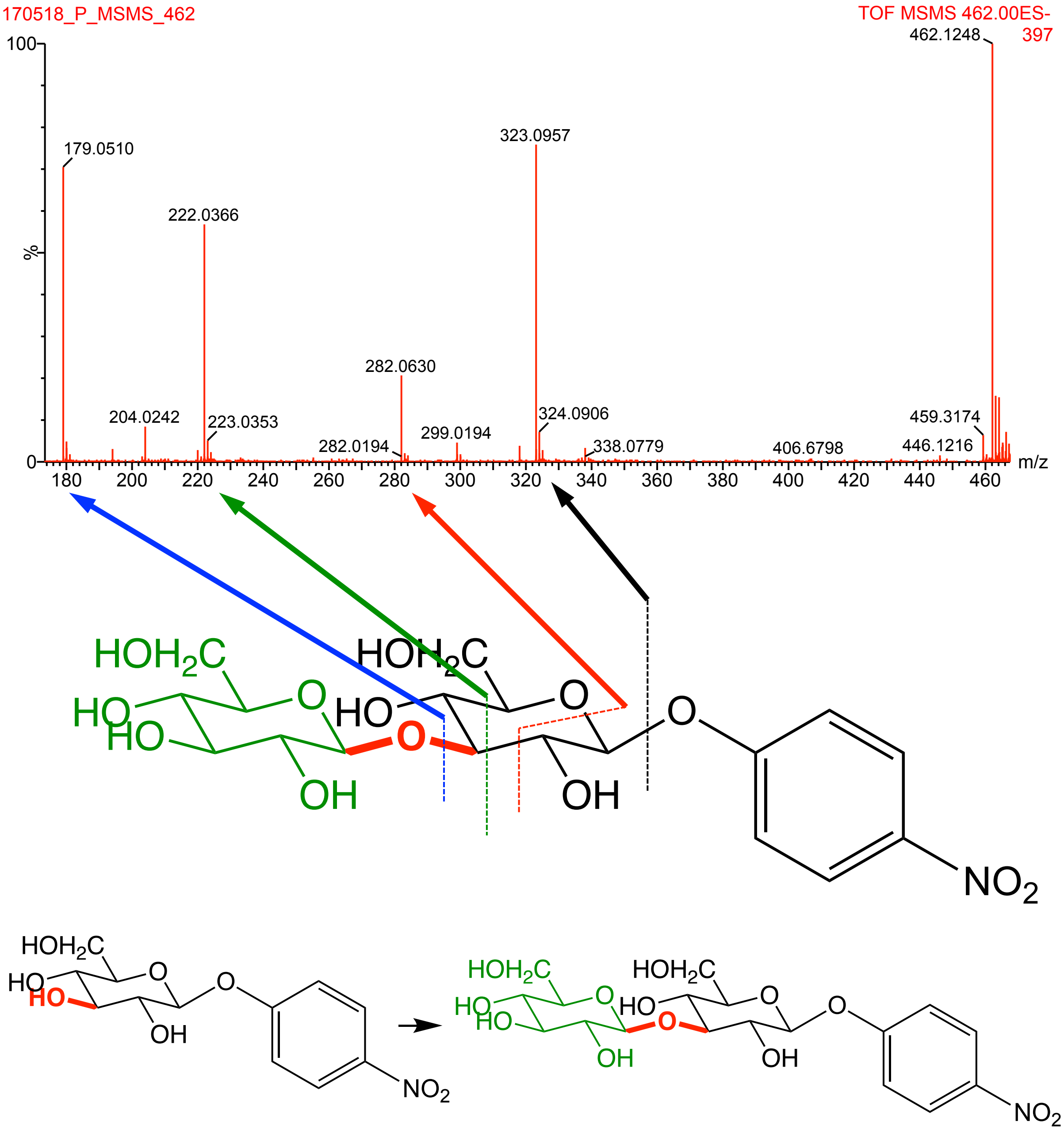

Supplementary Figure 14 MS and MS/MS of the reactions of 4-nitrophenyl β-D-glucopyranoside

(a) Direct MS of the reactions of 4-nitrophenyl β-D-glucopyranoside by UGT76G1. The reaction catalysed by 0.12 (1x) or 0.25 mg ml^{-1} (2x) of UGT76G1 was sampled and measured at the time course of 10 min, 2 hours and 12 hours. The two respective main negative ions derived from the substrate and product are labeled, which show the consumption of substrate and product yield is related to the enzyme concentration and the reaction duration. The product gains a mass of 162 Da to the substrate after the reaction, equivalent to a glucose addition

(b) MS/MS of the product. The negative ion of the product with m/z at 462.1 was specifically isolated and characterized. The fragmentation profile matches a di-saccharide group, which suggests the glucose addition occurs at the glucose of the substrate rather than at the 4-nitrophenyl ring.
