# Supplementary material for: Hydrophobic recognition allows the glycosyltransferase UGT76G1 to catalyze its substrate in two orientations
Source: Nat Commun. 2019 Jul 19;10:3214. doi: 10.1038/s41467-019-11154-4 (PMC6642264; doi:10.1038/s41467-019-11154-4)
Supplement: Supplementary file 3 — Reporting Summary [file 41467_2019_11154_MOESM3_ESM.pdf]

nature research

Corresponding author(s): Xiaofeng Zhu

Last updated by author(s): Jun 20, 2019

Reporting Summary

Nature Research wishes to improve the reproducibility of the work that we publish. This form provides structure for consistency and transparency in reporting. For further information on Nature Research policies, see Authors & Referees and the Editorial Policy Checklist.

Statistics

For all statistical analyses, confirm that the following items are present in the figure legend, table legend, main text, or Methods section.

n/a	Confirmed	
☐	☒	The exact sample size (*n*) for each experimental group/condition, given as a discrete number and unit of measurement
☐	☒	A statement on whether measurements were taken from distinct samples or whether the same sample was measured repeatedly
☒	☐	The statistical test(s) used AND whether they are one- or two-sided<br>*Only common tests should be described solely by name; describe more complex techniques in the Methods section.*
☒	☐	A description of all covariates tested
☒	☐	A description of any assumptions or corrections, such as tests of normality and adjustment for multiple comparisons
☒	☐	A full description of the statistical parameters including central tendency (e.g. means) or other basic estimates (e.g. regression coefficient) AND variation (e.g. standard deviation) or associated estimates of uncertainty (e.g. confidence intervals)
☒	☐	For null hypothesis testing, the test statistic (e.g. *F*, *t*, *r*) with confidence intervals, effect sizes, degrees of freedom and *P* value noted<br>*Give P values as exact values whenever suitable.*
☒	☐	For Bayesian analysis, information on the choice of priors and Markov chain Monte Carlo settings
☒	☐	For hierarchical and complex designs, identification of the appropriate level for tests and full reporting of outcomes
☒	☐	Estimates of effect sizes (e.g. Cohen's *d*, Pearson's *r*), indicating how they were calculated

Our web collection on statistics for biologists contains articles on many of the points above.

Software and code

Policy information about availability of computer code

Data collection: Mass spectrum data collected by using Masslynx v4.1, HPLC collected by Agilent 1260 Chemstation, Varioskan™ Flash multimode microplate reader used in the enzyme assays

Data analysis: X-ray data were analyzed by DIALS, CCP4 package (7.0) including COOT and Refmac and Phenix package (1.13). Skanlt software 2.4.3 RE for Varioskan Flash controlled the microplate reader and recorded the data.

For manuscripts utilizing custom algorithms or software that are central to the research but not yet described in published literature, software must be made available to editors/reviewers. We strongly encourage code deposition in a community repository (e.g. GitHub). See the Nature Research guidelines for submitting code & software for further information.

Data

Policy information about availability of data

All manuscripts must include a data availability statement. This statement should provide the following information, where applicable:

- Accession codes, unique identifiers, or web links for publicly available datasets
- A list of figures that have associated raw data
- A description of any restrictions on data availability

The coordinates and structure factors have been deposited in the Protein Data Bank under accession codes 6INF, 6ING, 6INH, 6INI. The The source data underlying the kinetic parameters in Table 1 are provided as a Source Data file. All relevant data are included in the paper, the Supplementary Information file or the Source data file.

Field-specific reporting

Please select the one below that is the best fit for your research. If you are not sure, read the appropriate sections before making your selection.

☒ Life sciences ☐ Behavioural & social sciences ☐ Ecological, evolutionary & environmental sciences

For a reference copy of the document with all sections, see nature.com/documents/nr-reporting-summary-flat.pdf

Life sciences study design

All studies must disclose on these points even when the disclosure is negative.

Sample size	Three independent experiments with similar results were widely used in vitro biochemical studies.
Data exclusions	No data were excluded.
Replication	All attempts at replication were successful.
Randomization	Randomization is not relevant to the majority of experiments of this study, because the purified individual protein samples are essentially identical and aren't grouped in in vitro activity assays and biochemical studies, but the proper controls were used to avoid any false positive results.<br>Randomization was used to assign the R-free flags in refinement of structure 6INF which has the highest resolution. To keep the validity of Rfree, the same assignment of R-free flags was used for the refinement of the other three structures 6ING,6INH and 6INI.
Blinding	Not applicable. This report involves only in vitro studies.

Reporting for specific materials, systems and methods

We require information from authors about some types of materials, experimental systems and methods used in many studies. Here, indicate whether each material, system or method listed is relevant to your study. If you are not sure if a list item applies to your research, read the appropriate section before selecting a response.

Materials & experimental systems

n/a	Involved in the study	
☒	☐	Antibodies
☒	☐	Eukaryotic cell lines
☒	☐	Palaeontology
☒	☐	Animals and other organisms
☒	☐	Human research participants
☒	☐	Clinical data

Methods

n/a	Involved in the study	
☒	☐	ChIP-seq
☒	☐	Flow cytometry
☒	☐	MRI-based neuroimaging
